# Repeat length in spinocerebellar ataxia type 4 (SCA4) predicts age at onset and disease severity

**DOI:** 10.1007/s00415-024-12600-0

**Published:** 2024-08-02

**Authors:** Andreas Dalski, Martje G. Pauly, Henrike Hanssen, Johann Hagenah, Yorck Hellenbroich, Christian Schmidt, Jassemien Strohschehn, Malte Spielmann, Christine Zühlke, Norbert Brüggemann

**Affiliations:** 1 Institute of Human Genetics, University Hospital Schleswig-Holstein, University of Luebeck, Ratzeburger Allee 160, 23538 Luebeck, Germany; 2 Department of Neurology, University Hospital Schleswig-Holstein, University of Luebeck, Ratzeburger Allee 160, 23538 Lübeck, Germany; 3 WKK Westkuestenkliniken, Heide, Germany; 4 Institute of Biology, University of Luebeck, Luebeck, Germany; 5 University of Kiel, Kiel, Germany

**Keywords:** Spinocerebellar ataxia, SCA4, Repeat expansion, Anticipation

## Abstract

**Background:**

Recently, an exonic GGC repeat expansion (RE) was identified by long-read genome sequencing in the *ZFHX3* gen, causing spinocerebellar ataxia type 4 (SCA4), a dominant form of ataxia with sensory neuropathy. However, the analysis of larger cohorts of patients remained demanding, resulting in a challenge to diagnose patients and leaving the question of anticipation in SCA4 unanswered.

**Objectives:**

We aimed to develop a GGC repeat test for clinical SCA4 screening and to apply this test to screen two large German SCA pedigrees and samples of unrelated patients collected over the last 25 years.

**Methods:**

We modulated a commercial GGC-RE kit (Bio-Techne AmplideX^®^ Asuragen^®^ PCR/CE FMR1 Reagents) with *ZFHX3*-specific primers and adapted PCR conditions. The test was applied to patients and 50 healthy controls to determine the exact repeat number. Clinical data were revised and correlated with the expanded allele sizes and an exploratory analysis of structural MRI was performed.

**Results:**

Repeat size, determined by our protocol for (GGC)_n_ RE analysis shows a strong inverse correlation between repeat length and age at onset and anticipation in subsequent generations. The phenotype also appears to be more strongly expressed in carriers of longer RE. Clinical red flags were slowed saccades, sensory neuropathy and autonomic dysfunction.

**Conclusion:**

Our protocol enables cost-effective and robust screening for the causative SCA4 RE within *ZFHX3*. Furthermore, detailed clinical data of our patients gives a more precise view on SCA4, which seems to be more common among patients with ataxia than expected.

**Supplementary Information:**

The online version contains supplementary material available at 10.1007/s00415-024-12600-0.

## Introduction

The spinocerebellar ataxias (SCAs) with autosomal dominant inheritance are a clinically and genetically heterogeneous group of neurological disorders characterized by isolated or predominant cerebellar ataxia with additional neurological manifestations [1]. Genetically, two major groups are defined: SCAs related to deletions, insertions, missense, nonsense or frameshift mutations in the corresponding genes, and SCAs caused by dynamic repeat expansions (RE), the most frequent type of mutations in inherited ataxias.

SCA4 (MIM 600223), a spinocerebellar ataxia type with sensory axonal neuropathy, was previously linked to chromosome 16q22.1 in a large kindred of Scandinavian origin residing in Utah [2]. In 2003, a second SCA4 family with Scandinavian ancestor was reported from Northern Germany [3].

Recently, a heterozygous GGC repeat expansion coding for a polyglycine (polyG) within the zinc finger homeobox 3 gene (*ZFHX3;* MIM 104155) was identified in SCA4 pedigrees [4–7] using long-read genome sequencing (LR-GS), *ZFHX3* encodes a transcription factor with multiple homeodomains and zinc finger motifs. *ZFHX3* is involved in various biological processes, including myogenic and neural cell differentiation and tumorigenesis. Loss-of-function mutations in *ZFHX3* cause syndromic intellectual disability [8] with variable phenotypes as autism spectrum disorder, facial features, relative short stature or brachydactyly.

Since long-read sequencing is still an expensive technology with difficulties to produce adequate coverage over the repeat, we aimed to develop a robust, reliable and cost-effective PCR-protocol to amplify and analyze the repeat length from genomic DNA on the basis of a commercial PCR kit. We applied this method to two large kindreds and nine unrelated patients to identify potential correlations of repeat size, age of onset (AOO) and disease severity. In addition, we present detailed clinical information of patients of one of these kindreds and results from prospectively collected structural MRI images.

## Patients and methods

### Patients

All patients were tested negative for SCA1, SCA2, SCA3, SCA6, SCA7, SCA8, SCA12 and SCA17 repeat expansions. Repeat expansions in 34 candidate genes located in 16q22.1 were excluded[9].

### Family I

**Fig. 1:**
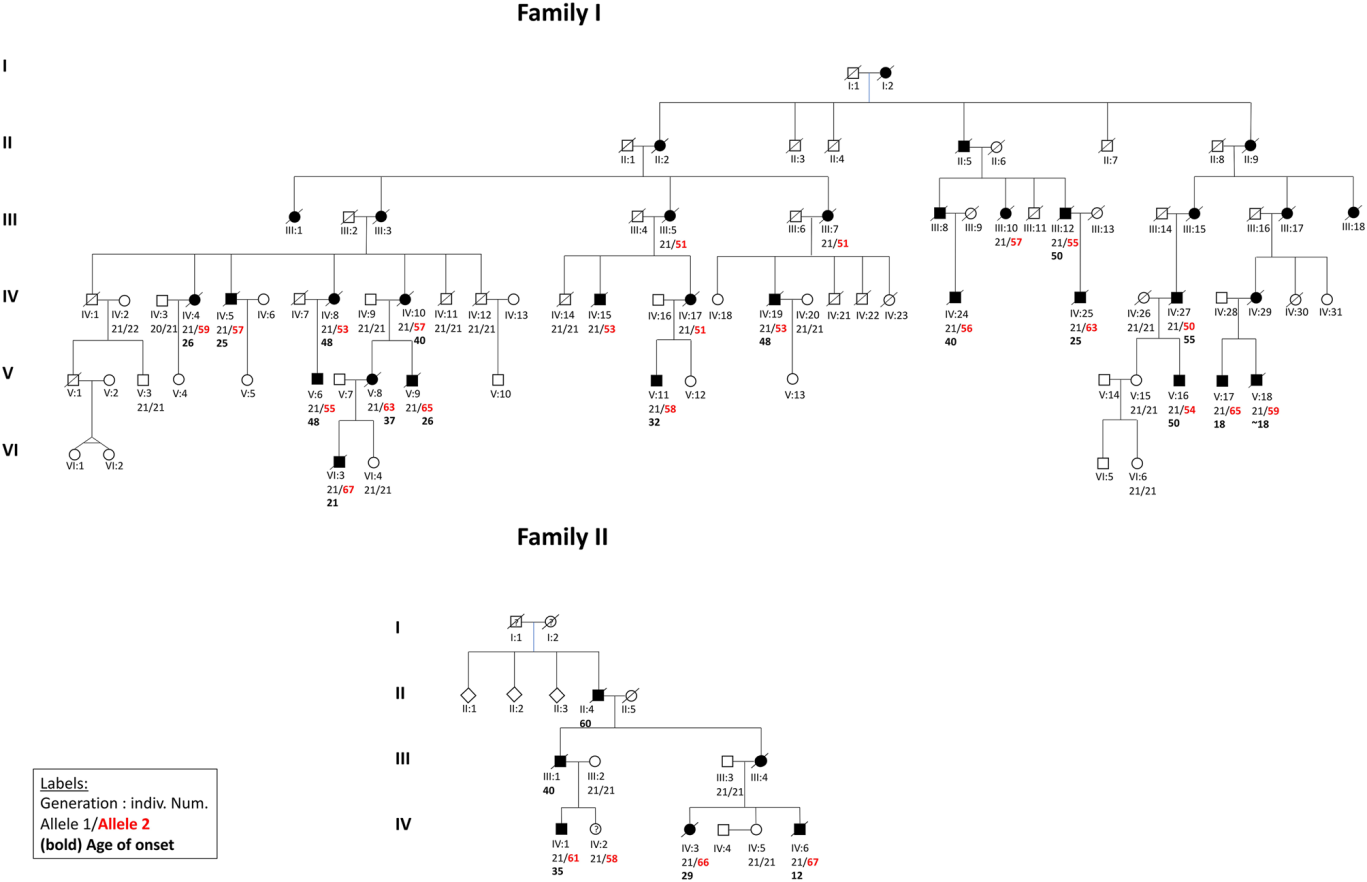
Family I and II; Standard symbols were used. Black symbols represent individuals with cerebellar ataxia with sensory and autonomic neuropathy. Individual indices, allele lengths and age of onset (AOO) are given beneath pedigree symbols. *Red* expanded allele sizes

Six-generation SCA family from Northern Germany with 33 affected individuals. Twelve of these were examined by movement disorders neurologists between 1991 and 2023 at our Departments of Human Genetics and Neurology. For two patients of family I (V 17 and V 18) detailed clinical information was provided from neurologists of other hospitals. During routine clinical visits, imaging (MRI and cCT), nerve conduction studies (NCS), and acoustic (AEP), visual (VEP) and sensory evoked potentials (SEP) as well as routine EEG was performed (Fig. 1 and Table 1).

**Table 1:** Family I: clinical datasets

Patient (family I)	III 12	IV 5	IV 8	IV10	IV 19	IV 25	V 6	V8	V 16	V17	V 18	VI 3
Exp. repeat size	55	57	53	57	53	63	55	63	54	65	59	67
Sex	Male	Male	Female	Female	Male	Male	Male	Female	Male	Male	Male	Male
Initial symptom	Unsteady Gait, Dysarthria, Dysphagia		Unsteady gait		Dysarthria	Unsteady gait	Unsteady gait		Unsteady gait	Reduced sensation	Unsteady gait	Unsteady gait
AOO	49	25	48	40	48	25	48	37	50	18	18	21
AE (last)	58	57	54	82	63	33	51	55	54	42	47	36
Death (age)	59										47	36
Gait ataxia	Yes	Yes	Yes	Yes	Yes	Yes	Yes	Yes	Yes	Yes	Yes	Yes
Limb ataxia	Yes		Yes	Yes	Yes	Yes	Yes		Yes	Yes	Yes	Yes
Romberg test	Yes		Yes	Yes	Yes	Yes	Yes		Yes	Yes		Yes
Dysarthria	Yes		No	Yes	Yes	Yes	Yes		No	Yes	Yes	Yes
Reduced sensation	Yes		No	No	Yes	Yes	No		No	Yes	Yes	No
Impaired propriozeption	Yes				Yes	Yes	Yes		Yes	Yes	Yes	Yes
Absent ankle jerks											YES	
Areflexia	Yes		Yes		Yes	Yes	Yes		Yes	Yes		yes
Extensor plantar reflexes	No		No	No	No	Yes				Yes	No	No
Spasticity	No		No	No		No	No		No		No	No
Weakness	No		No							Yes		
Nystagmus	No		No			No			No	No		
Slow horizontal saccades	Yes				Yes	Yes	Yes		Yes	Yes		Yes
Hypometric saccades												Yes
Incomplete bladder emptying			No			No	Yes			Yes	Yes	Yes
Constipation		Yes								yes		
Orthostatic Hypotension		Yes				No	No		no	yes		
Faciolingual fasciculations		Yes				Yes						
Head tremor	Yes		No			Yes						
Dystonia						Cervical	Cervical				Limbs	
Bradydisdochokinesia	Yes		Yes		yes		Yes		Yes	Yes	Yes	
EEG	Normal		Slight general slowing									
Neuroimaging atrophy	MRI: CB, medulla oblongata, SC		CT: CB	MRI: CB, SC		MRI: CB	MRI: CB			MRI: CB		MRI: CB
AEP	Normal		Normal									
VEP			Normal									
SEP	Reduced (UL + LL)		Absent (UL + LL)				Absent (UL + LL)			Reduced (UL < LL)		Absent (LL)
NCS												
Motor	Normal		Normal				Normal			Normal		Reduced
Sensory (SNAP)	Absent (UL + LL)		Absent (UL + LL)				Absent (UL + LL)			Abenst (UL + LL)		Absent (UL + LL)
ENG	slow saccades, impaired optokinetic nystagmus											

*AOO* Age of onset, *AE* Age of clinical examination, *CB* Cerebellum, *SC* Spinal cord, *UL* Upper limbs, *LL* Lower limbs, *EEG* Electroencephalography, *AEP* Acoustic evoked potentials, *VEP* Visual evoked potentials, *SEP* Somatosensory evoked potentials, *NCS* Nerve conduction study, *ENG* Elektronystagmography

### Family II

Four generation family with six affected individuals showing spinocerebellar ataxia and sensory axonal neuropathy (Fig. 1).

### Unrelated patients

Of 368 single index cases with ADCA (autosomal dominant cerebellar ataxia), 9 unrelated (including 1 pair of twins) patients (Table 2) were identified by a *CALB2* SNP (rs1362504041) which segregated with SCA4 in all affected individuals in family I.

**Table 2:** Unrelated SCA4 patients

Patient:	male	female	Allele 1:	Allele 2:	AOO
P1	x		21	53	Not reported
P2		x	21	50	43
P3		x	21	45	55
P4		x	21	51	46
P5		x	21	56	40
P6^*^	x		21	50	Not reported
P7^*^	x		21	50	Not reported
P8			21	**68**	16
P9	x	x	21	**44**	ca. 60
P10	x		21	56	< 40

Of 368 single index cases with ADCA (autosomal dominant cerebellar ataxia) nine patients were identified by a *CALB2* SNP (rs1362504041) which segregates with SCA4 in all affected individuals in family I

^*^Twins; bold is smallest and largest pathological alleles

### PCR amplification and fragment analysis

DNA samples from peripheral blood extracted with different protocols were collected over the last 3 decades and diluted to an average concentration of 50 ng/µl. PCR was performed on the basis of a commercial diagnostic FMR1-Kit (Bio-Techne AmplideX^®^ Asuragen^®^ PCR/CE FMR1 Reagents). We replaced the gene specific kit primers by the following *ZFHX3*-primers:

SCA4-fwd: gttccaaaggtgcagtacaagttggtctg.

SCA4-rev: gttctgtgtttgtgcaaggccgactcgag (5 ‘-6FAM labeled).

Third primer for the CGG-repeat was used as included in the kit. PCR product size for the most abundant GGC/CCG-repeat is 299 bp representing 21 repeat units.

The PCR protocol was adapted to the following times and temperatures:

Initial denaturation at 95 ℃/5 min followed by 20 cycles of 97 ℃/ 35 s, 62 ℃/35 s and 68 ℃/2 min.

Additional 20 cycles with a 20 s increment per cycle were performed with same times and temperatures and a final elongation step at 72 ℃ for 10 min.

**Fig. 2:**
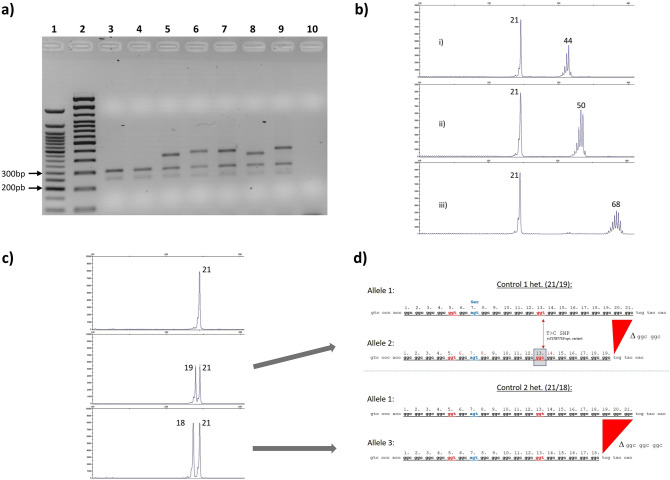
**a** 2% Agarose gel: PCR products of different patients (Family I); lane 1 50 bp-Ladder (NEB), lane 2 100 bp-Ladder (Invitrogen); lane 3 and 4 control samples, lane 5 patient IV:10; lane 6 patient V:17; lane 7 patient V:8; lane 8 patient III:7; lane 9 patient VI:3; lane 10 negative PCR control; **b** Capillary gel electrophoresis of (i) smallest, (ii) intermediate and (iii) longest pathological allele in our cohort. Numbers indicate allele repeat size: (21 = common normal allele); **c** Capillary peaks with repeat sizes of three control samples. Upper lane homozygous 21 repeats, middle lane heterozygous 19 and 21 repeats, bottom lane heterozygous 18 and 21 repeats. **d** Sequences of heterozygous controls with 21, 19 and 18 repeat alleles, respectively

PCR-products were checked on a 2% agarose gel (Fig. 2a) and finally separated on a capillary following the standard procedure as described for the FMR1-kit (Fig. 2b, c). Sanger-sequencing was performed according to standard protocols (Fig. 2d and Online Resource 1, Fig. 1).

### Structural neuroimaging

Structural brain MRI images for three *ZFHX3* variant carriers from family I (IV 10, IV 8 and V 16) were acquired in 2010 on a 3.0 T whole-body scanner (Philips, Achieva, The Netherlands) using a 3D T1- weighted gradient echo sequence (echo time = 3.7 ms; repetition time = 8.1 ms; flip angle = 8°; voxel size = 1 × 1 × 1 mm^3^). For each variant carrier, an age-matched healthy control group was examined on the same scanner. IV 10 (female) was 71 years at the time of examination with a disease duration of 24 years. The control group comprised four females and nine males with a mean age of 71.0 years (standard deviation 2.29). IV 8 (female) was 69 years with a disease duration of 30 years at the time of the examination. The control group contained 6 females and 11 males with a mean age of 68.9 years (standard deviation 3.27). V 16 (male) was 46 years at the time of examination and did not experience any symptoms until the age of 50. The control group comprised five females and six males with a mean age of 47 years (standard deviation 3.84). T1-weighted images were brought to MNI space using the standard pipeline of the CAT12 toolbox[10] in SPM 12 (University College London, Wellcome Trust Centre for Neuroimaging; http://www.fil.ion.ucl.ac.uk/spm)) in Matlab (MathWorks, Natick, MA). A cerebellar volumetry was performed using the SUIT atlas[11]. The occipital pole was chosen as a reference volume as no changes are expected in this region. As there was no hypothesis regarding hemispheric differences total volume of each cerebellar lobule was calculated. After scaling volumes to the total intracranial volume, the data was z transformed for comparability. A patient’s z score of was considered significant if higher or lower than 1.96 (95% confidence interval).

### Statistics

Normality of data distribution was tested using the Shapiro–Wilk test with data considered to be normal distributed if the p-value is larger than 0.05. For correlation of repeat size and AOO, the Pearson correlation coefficient was calculated. For demographic, MRI and clinical data, a descriptive analysis without formal statistics was performed. Data are reported as mean ± standard deviation.

## Results

### PCR fragment analysis

The adapted PCR protocol allowed us to screen all DNA samples of family members and individual patients. Repeat length differences of control samples and patients could be seen on agarose control gels, already (Fig. 2a). Capillary gel electrophoresis of PCR products allowed us to determine the correct repeat sizes for all tested patients and controls (Fig. 2b and c).

The largest expansion in our cohort is composed by 68 GGC triplets in patient 8 (Table 2) with an AOO of 16 years, the shortest pathological repeat in our collective turned out to be 44 repeats with an AOO of about 60 years, respectively (Table 2; P9).

Sanger sequencing of control and patient samples could confirm the repeat lengths and structures. Common normal alleles are composed of 21 triplet units*,* whereas heterozygous controls with alleles of 18/21 or 19/21 repeats are also present. The normal 21 allele typically has a structure build of 6 and 14 glycine coding elements interrupted by an AGT serine coding triplet at position 7 (Fig. 2d). At position 5 and 13, the GGC stretches are interrupted by synonymous glycine coding GGT triplets. Length differences are due to the loss of two or three GGC units, respectively. Interestingly, the 19 repeat allele (allele 2) misses the synonymous GGT interruption at position 13.

The same sequencing approach was applied to the mixture of normal and expanded alleles in affected patients. By extracting separated PCR bands from the agarose gel, we were able to generate clear signals for the smaller normal allele and heterozygous signals for expanded alleles at the nucleotide positions, where the above-mentioned interruptions are located (Online Resource 1, Fig. 1). These results underline the fact that all interruptions are lost in expanded alleles, indicating their potential role as stabilizing elements in normal alleles.

**Fig. 3:**
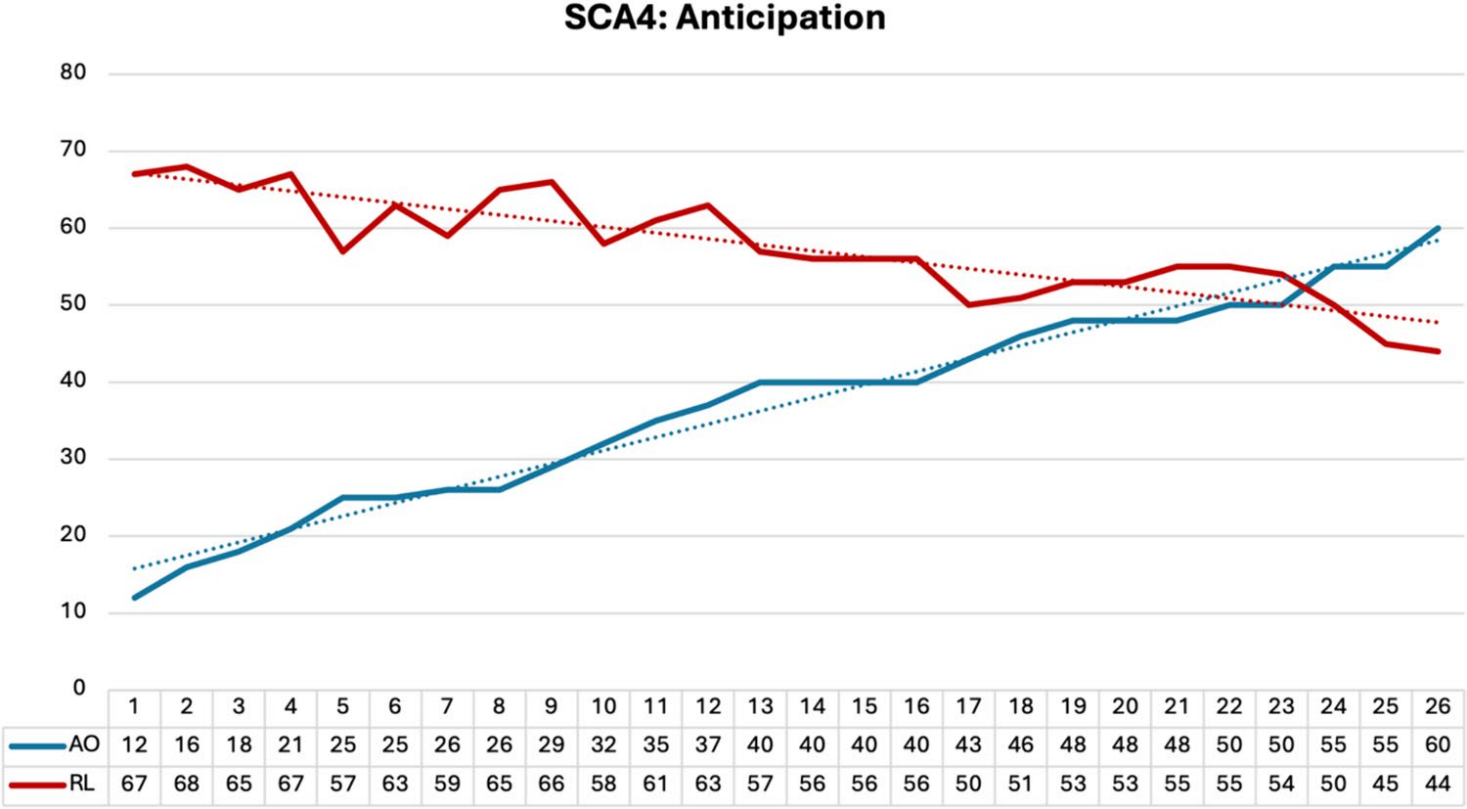
Anticipation in SCA4 in subsequent generations of families I and II and inverse correlation between age of onset (AOO) and repeat length (RL) of 26 SCA4 patients

Within the two families, we observed (i) strong anticipation (Fig. 3) from generation to generation with relatively small differences in repeat lengths, and (ii) a negative correlation between the repeat length of the longer allele and AOO (Pearson correlation coefficient *R* =  − 0.901, *R*^2^ = 0.812, *p* < 0.001) explaining of the variability of AOO. The data were normally distributed (Shapiro Wilk test statistic 0.981, *p* = 0.889). In family I we observed a decrease of AOO from 50 to 21 years from generation III to generation VI and an increase of the pathological repeat size from a minimum of 51 to a maximum of 67 repeats, respectively.

Furthermore, nine non-related patients (Tab. 2), with dominant ataxia, turned all out to be carriers of a *ZFHX3* expanded allele where two patients carry expansions of only 44 and 45 GGC repeats.

### Clinical characterization

The clinical phenotype of the members of family I is described in the following case reports and in Table 1.

#### III 12 (55 repeats)

This male patient first noticed an unsteady gait, slurred speech and swallowing problems at age 49 years. The symptoms progressed slowly and at the age of 58 he presented with marked dysarthria, areflexia, and severe gait ataxia. NCS confirmed an axonal polyneuropathy. The MRI revealed an atrophy of the cerebellum, medulla oblongata, and cervical spinal cord. The patient died of an unknown cause at the age of 59 years.

#### IV 8 (53 repeats)

This female patient developed an unsteady gait at age 48 years with occasional twisting of her ankles. At the age of 54 years, examination revealed no dysarthria, but areflexia and gait ataxia. A CT scan showed mild cerebellar atrophy.

#### IV 10 (57 repeats)

This female patient developed first symptoms at the age of 40 years. At the age of 82, she presented to the hospital due to a stroke. Regardless of the stroke she showed dysarthria, limb ataxia and a depressive mood.

#### IV 19 (53 repeats)

This male patient developed dysarthria at the age of 48 years followed by progressive cerebellar ataxia with an inability to stand unaided at the age of 61 years. Aged 63, he presented with difficulties to sit without losing balance and falls from the wheelchair.

#### IV 25 (63 repeats)

This male patient noticed an unsteady gait at age 25 years as well as mild slurring of his speech. At age 27, he showed mild dysarthria and mild cervical dystonia. The MRI showed distinct cerebellar atrophy. Gait ataxia progressed over time and the patient suffered from frequent falls. He started to use a walker at around the age of 32 years. Dysarthria was also progressive as well as tingling in finger and hands. At the examination at age 33 years, dysarthria and gait as well as limb ataxia have continuously progressed. For videos of patient see Online Resource 2 (Video 1).

#### V 6 (55 repeats)

This male patient noticed an unsteady gait at the age of 48 years. At age 51 years, he presented with slow horizontal saccades, mild dysarthria, limb and gait ataxia, as well as areflexia. An MRI showed cerebellar atrophy and NCS confirmed sensory polyneuropathy of the upper and lower extremities independent of the nerve length.

#### V 16 (54 repeats)

This male patient noticed an unsteady gait at the age of 50 years with swaying to both sides as well as reduced fine motor skills. At age 53 years, he presented with lower limb and gait ataxia, reduced vibration sense and areflexia. His gait ataxia progressed over the next 1.5 years to the need to use a walker outside the home. Clinical examination at the age of 54 years revealed progressive limb and gait ataxia, but still no dysarthria.

#### V 17 (65 repeats)

This male patient noticed reduced sensation at the finger tips and feet at the age of 18 years. He developed an unsteady gait and slurred speech. Aged 27, he started urinary self-catheterization due to bladder emptying disorder. At the examination at age 29 years, he presented with slow horizontal saccades, dysarthria, gait ataxia and sensory neuropathy. An MRI showed atrophy of the cerebellum and NCS confirmed severe sensory neuropathy. All symptoms progressed over time and the patient additionally suffered from orthostatic hypotension. At around age 40, he started using a wheelchair. Aged 42 years, he presented with severe dysarthria, oromandibular dystonia, ocular apraxia with head movements while generating saccades. Ataxia had also significantly worsened.

#### V 18 (59 repeats)

This male patient noticed first symptoms aged 18 years. At the age of 29 years, he presented with gait ataxia, areflexia, as well as hypesthesia and impaired proprioception in the lower limbs. He developed a progressive gait disorder with subsequently frequent falls, dysarthria and bladder emptying problems. When he was admitted to a hospital due a severe cervical spinal stenosis at the age of 47 years, he showed gait and limb ataxia, hypesthesia of both legs as well as reduced sense of vibration and proprioception. Although the neurosurgical operation was successful, he developed acute cardiac failure and pneumonia leading to death at age 47 years.

#### VI 3 (67 repeats)

This male patient noticed an unsteady gait at age 21 years, followed by an impairment of his fine motor skills. At around 22 years of age, he noticed a slurred speech. At 24 years, bladder emptying disorder was diagnosed. Three years later, he presented with mild dysarthria, saccadic smooth pursuit, mild ataxia and areflexia at the lower limbs, and an unsteady gait. NCS revealed sensory neuropathy. An MRI demonstrated cerebellar atrophy without structural abnormalities of the spinal cord. Upon his examination at the age of 26 years, limb ataxia was now also present in the upper limbs and the patient showed slowed saccades. Gait ataxia progressed over the next 2 years with occasional falls, while dysarthria did not worsen. At the age of 36, he developed dysphagia which was rapidly progressive thereafter leading to food aspiration and subsequent pneumonia with the need manual ventilation. Diagnostic workup revealed atony and lack of motility of the esophagus. After restriction of further invasive therapy, the patient died at age 36 years. For video of the patient, see Online Resource 3 (Video 2).

Only limited information on patients IV 5 and V 8 was available (see Table 1).

In summary, patients initially manifested with a gait disorder due to a combination of cerebellar ataxia and sensory neuropathy. Both cerebellar ataxia and sensory neuropathy seem to have equally contributed to the phenotype and impairment of patients’ daily life. Autonomic dysfunction occurred only later in the disease and was clinically relevant only in patients with a severe manifestation. Disease progressed noticeable over a short amount of time, especially in patients with longer repeats, sometimes resulting in premature death of the patient.

### MRI analysis

Cerebellar volumetry revealed widespread gray matter atrophy in the two symptomatic *ZFHX3* variant carriers but no significant atrophy in the presymptomatic carrier four years before his symptom onset (Online Resource 1, Fig. 2). Atrophy was present in most parts of the cerebellum including not only part of sensorimotor regions (lobules VI and VIII a/b) but also lobules associated with higher tasks (lobule VI and Crus I (language and verbal working memory), lobule VI (spatial tasks), lobules VI, VIIb and Crus I (executive functions) and lobule VI and Crus I (emotional processing). The anterior lobe (lobules I–V—sensorimotor tasks) did not show atrophy [12]. Neither did neurodegeneration affect cerebellar white matter in the investigated patients.

## Discussion

*ZFHX3* is the first published gene with a coding GGC repeat translated into a polyglycine stretch in the active protein, in contrast to GGC repeats in the 5’UTR *of FMR1* or *NOTCH2NLC*. The common normal allele with 21 repeats consists of 18 GGC triplets with two inserted GGT units at position 5 and 13, and one serine coding AGT triplet at position 7[5]. This normal allele was found in combination with an expanded allele (ranging from 44 to 68 repeat units) in all of our patients. In our control samples, we identified normal alleles of 18, 19, and 22 repeats. Remarkably, the 19 repeats allele misses the second GGT interruption at position 13. It is a long-discussed issue, if interruptions within the repetitive sequence as seen e.g. in SCA1 (CAT) and SCA2 (CAA) have a stabilizing effect, preventing the expansion of a normal allele to expand. Our sequencing data underline this hypothesis, because all expanded SCA4 alleles are free of the above-mentioned interruptions.

**Fig. 4:**
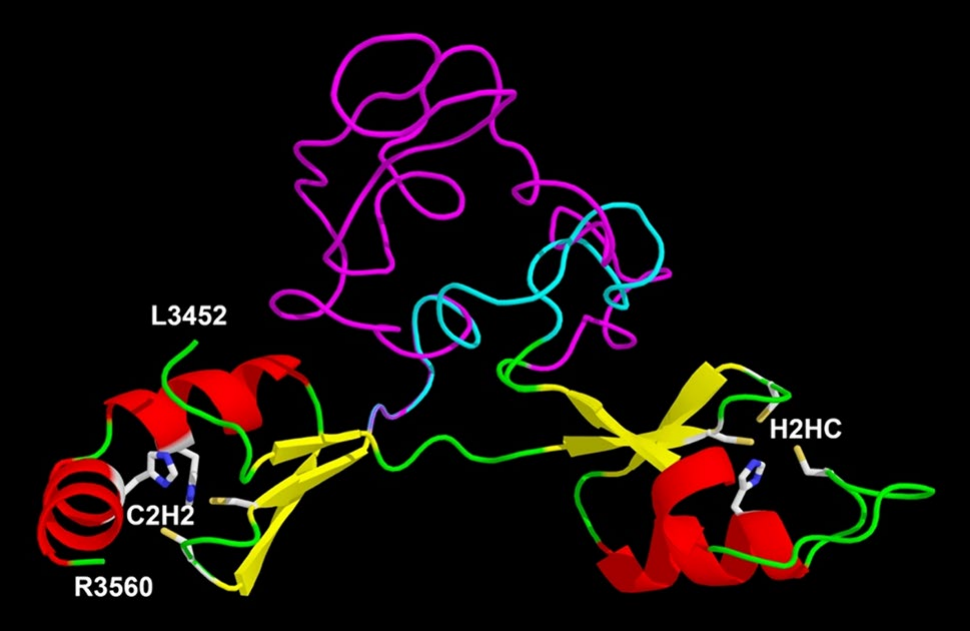
Homology models of region 3452–3560 of the human ZFHX3 protein (GenBank: AAC14462.1) and a variant containing 47 additional glycine residues

Compared to e.g. SCA2 and SCA7, where expansions may rise up to more than 100 repeats, the range of pathological expansion in *ZFHX3* is rather small (42 to 74^5^ and 44 to 68 in our cohort). First computational protein modelling for expanded sequences (Fig. 4) showed that the polyglycine stretch seems to be a linking element between two zinc finger motives. Zinc fingers are essential for protein function in binding promotors of genes involved in nervous system development [8]. This leads to the assumption that only a limited number of glycine residues at this position are tolerated and that the pathological gain of function rises with the increasing number of glycines. This might be an explanation for the clear anticipation found in our pedigrees.

The models were calculated using the Swiss-Modell service (https://swissmodel.expasy.org/) with the AlphaFold DB model of A0A0S7IW54_9TELE (gene: ZFHX3, organism: *Poeciliopsis prolifica*) as template and visualised using the Pymol program.

Helices are shown in red, ß-strands in yellow and loops in green. The glycine-rich stretch of ZFHX3 variant AAC14462.1 is coloured turquoise, the extended glycine stretch containing 47 additional glycine residues is shown in magenta.

The side chains of the predicted Zn-coordinating residues are shown. For the C2HC zinc finger (right side of the figure) C3467, C3470, H3483, C3488 and for the C2H2 zinc finger C3531, C3534, H3547 and H3553 (all numbers refer to the sequence of AAC14462.1).

The repeat lengths correlate well with the AOO in our patients (16 to 60 years). In our view, the repeat size determination of a larger number of symptomatic individuals is needed to evaluate the SCA4 disease spectrum in relation to the expansion. Furthermore, our findings show that SCA4 is not a clear late-onset disorder but also affects younger patients (early onset).

Interestingly, loss-of-function mutations in *ZFHX3* are associated with variable intellectual disability and autism spectrum disorder, recurrent facial features, relative short stature, or brachydactyly^8^, phenotypes not seen in SCA4 patients. *ZFHX3* is intolerant to protein-truncating variants (PTV) or partial deletions. Remarkably, no PTVs are observed at the C-terminus of *ZFHX3*, potentially C-terminal mutations escape NMD (nonsense-mediated mRNA decay). In addition, compound heterozygous *ZFHX3* variants were identified in children with partial epilepsy [13].

The nine unrelated SCA4 patients were selected by the *CALB2* SNP in 368 individuals with dominant ataxia (2.45% of unclassified ADCA patients in our cohort). In addition, the *CALB2* SNP co-segregates with SCA4 in family 1 and 2 and is very rare in controls (0 in 600 samples, unpublished results, nucleotide C is evolutionary conserved). This finding points to a common founder for SCA4.

In comparison to LR-GS, the simple PCR assay developed in our lab will overcome some technical difficulties. Particularly, independent of the DNA isolation method and age of sample, all samples turned out to be suitable for PCR amplification, a clear advantage over the modern sequencing methods. Furthermore, the PCR is appropriate to test the prevalence of SCA4 in larger cohorts and populations as well as to validate the obvious inverse correlation between repeat length and AOO. Possibly, the method used in our lab may be adapted to further genes with GGC repeats like *NOTCH2NLC*. We recommend our protocol as a cost-effective alternative to long read sequencing whole genome applications in case of predictive testing in a SCA4 positive family, and in individual cases with typical clinical signs and a reasonable suspicion of a diagnosis of SCA4. Furthermore, based on our protocol, it is our goal to develop a PCR-based neuro-panel for testing all (SCA) difficult repeat sequences on a nanopore platform in parallel.

The clinical presentation of the members of Family I with gait ataxia, dysarthria and sensory neuropathy is in line with the previous reports of patients with SCA4 [2, 5]. The sensory neuropathy manifested clinically with a severe impairment of proprioception, while superficial sensation was perceived as normal by patients, contributing to gait ataxia especially when deprived of visual aid. Upon NCS, the neuropathy was purely sensory in most cases, with reduced or absent sensory evoked potentials and absent sensory nerve action potentials in both, the lower but also the upper extremities. The observation of a sensory neuropathy independent of the length of the nerves suggests a ganglionopathy rather than an affection of peripheral sensory fibers as the underlying cause. A pathology of sensory ganglia has previous been demonstrated in other monogenic ataxias such as *RFC1*-associated cerebellar ataxia, neuropathy, vestibular areflexia syndrome (CANVAS) [14]. In *RFC1*-associated ataxia, an additional pathology of the vestibular and trigeminal ganglia is present [15], raising the question of a similar affection in SCA4. The vestibular function should thus be considered and prospectively investigated in future phenotyping studies in SCA4, using calorimetry and quantitative head impulse testing. In addition to the sensory impairment, patients with longer repeat lengths clearly exhibited autonomic involvement with orthostatic hypotension, bladder emptying problems resulting in the necessity of self-catheterization at a young age, and constipation. Another distinct feature in most of our patients was slowness of saccades, which was described in six patients. Due to the retrospective character of gathering the clinical information, electronystagmography was only performed in one patient (III 12), confirming slowing of saccades and impaired optokinetic nystagmus. Some patients used ‘head saccades’ while looking to the left or right which could be due to slowness of saccades. These head movements are also observed in patients with ocular apraxia where fast head turning maneuvers facilitate the generation of horizontal saccadic eye movements. Whether an impaired saccade initiation as a marker of ocular apraxia is additionally present in SCA4 remains to be elucidated in future studies.

Exploratory cerebellar volumetry in two patients showed widespread cerebellar gray matter atrophy affecting not only regions associated with sensorimotor tasks but also higher cognitive tasks despite the pure clinical motor phenotype described so far. Further specific testing especially of executive functions is warranted in future investigations. The sparing of white matter contrasts with a recent study examining a small heterogenous group of SCA patients (SCA2, SCA3, SCA6 and SCA 28). Here, extensive white matter loss was present in the cerebellar hemispheres and vermis extending to the brainstem [16]. Of course, both studies are limited by the low case numbers so larger controlled quantitative MRI studies are needed to investigate whether the sparing of white matter is a unique sign of SCA4 patients. Cerebellar volumetry revealed no significant atrophy in a prodromal *ZFHX3* variant carrier 4 years prior to symptom onset. Whereas quantitative MRI data on other prodromal SCA patients are scarce, a non-quantitative analysis of non-ataxic SCA2 variant carriers showed light cerebellar atrophy in 15 out of 24 individuals (62.5%) including the hemispheres and vermis [17].

Taken together, longer repeats seem to not only lead to an earlier AOO, but also to more severe clinical features, autonomic dysfunction and a more rapid disease progression. Patients in Family I with a high repeat number had severe ataxia, as well as autonomic dysfunction resulting in severe dysphagia due to an atony and lack of motility of the esophagus, resulting in an early death at age 36 years of the family member with the highest repeat number (VI 3, 67 repeats). To determine the exact correlation between repeat number and disease severity, further longitudinal studies and the use of scores such as the Scale for the assessment and rating of ataxia (SARA) following the example of prospective studies in different other SCAs [18–20] will be needed.

Due to the retrospective character of the clinical part of this study, there are some limitations: while patients were evaluated at different time points, not all examinations such as nerve conduction studies and MRI were performed. Also, not all clinical information was available in all patients in the same extend with quantifying score mostly not available.

Our results and the growing number of SCA4 reports suggests that this ataxia is more common than expected previously. Therefore, we suggest to include the SCA4 repeat in diagnostic procedures at least in dominant ataxia cases.

Overall, our development of a reliable and cost-efficient method to detect GGC-repeat expansion in the *ZFHX3* gen and determine the repeat length will help to identify more patients with SCA4. This will hopefully allow prospective assessment of nerve conduction studies and clinical development to further investigate the phenotype-genotype correlations and to evaluate potential further symptoms such as vestibular dysfunction.

## Supplementary Information

Below is the link to the electronic supplementary material.

Supplementary file1 (DOCX 472 KB)

Supplementary file1 Video 1 Video of patient IV 25 at age 33 years showing speech, holding arms in front, finger to nose test, finger to finger test, heel knee test, alternating hand movements, smooth pursuit, saccades, standing up and gait (MOV 128124 KB)

Supplementary file1 Video 2 Video of patient VI 3 at age 27 year showing holding arms in front, finger to nose test, finger following test, saccades and gait (MP4 154905 KB)

## Data Availability

The data that support the findings of this study are available from the corresponding author upon reasonable request.
