# Supplementary material for: Repeat length in spinocerebellar ataxia type 4 (SCA4) predicts age at onset and disease severity
Source: J Neurol. 2024 Aug 2;271(9):6289–300. doi: 10.1007/s00415-024-12600-0 (PMC11377680; doi:10.1007/s00415-024-12600-0)
Supplement: Supplementary file 1 — Supplementary file1 (DOCX 472 KB) [file 415_2024_12600_MOESM1_ESM.docx]

**Supplement**

**Repeat length in spinocerebellar ataxia type 4 (SCA4) strongly predicts age at onset and disease severity**

Andreas Dalski, PhD, Martje G. Pauly, MD, Henrike Hanssen MD, Johann Hagenah, MD, Yorck Hellenbroich, MD, Christian Schmidt, PhD, Jassemien Strohschehn, Malte Spielmann, MD, Christine Zühlke, PhD, Norbert Brüggemann, MD.

**Correspondence to:** Prof. Dr. med. Norbert Brüggemann, Department of Neurology, University Hospital Schleswig-Holstein, University of Luebeck, Ratzeburger Allee 160, 23538 Lübeck, Germany; E-mail: [Norbert.Brueggemann@uksh.de](mailto:Norbert.Brueggemann@uksh.de)

and / or

Prof. Dr. rer. nat. Christine Zühlke, Institute of Human Genetics, University Hospital Schleswig-Holstein, University of Luebeck Ratzeburger Allee 160, 23538 Luebeck, Germany; E-Mail: [Christine.Zuehlke@uksh.de](mailto:Christine.Zuehlke@uksh.de)

**Journal of Neurology**

**Supplementary Figures**

**
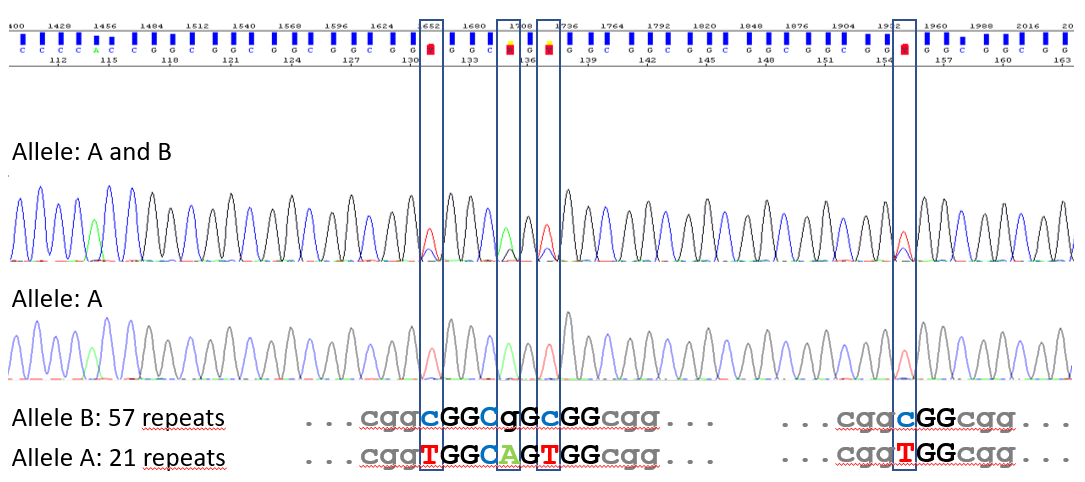
**

Supp. Fig 1: Sanger sequencing of PCR products of normal and expanded alleles separated by agarose gel electrophoresis and gel extraction. Upper lane shows a mixture of normal (21 repeats) and expanded allele (57 repeats) of patient IV:10 (family I). Lower lane shows sequence of the common normal allele (same patient). Sequence of normal allele with typical repeat interruptions (framed in boxes). Expanded repeat shows signals with loss of heterogeneity at these positions.


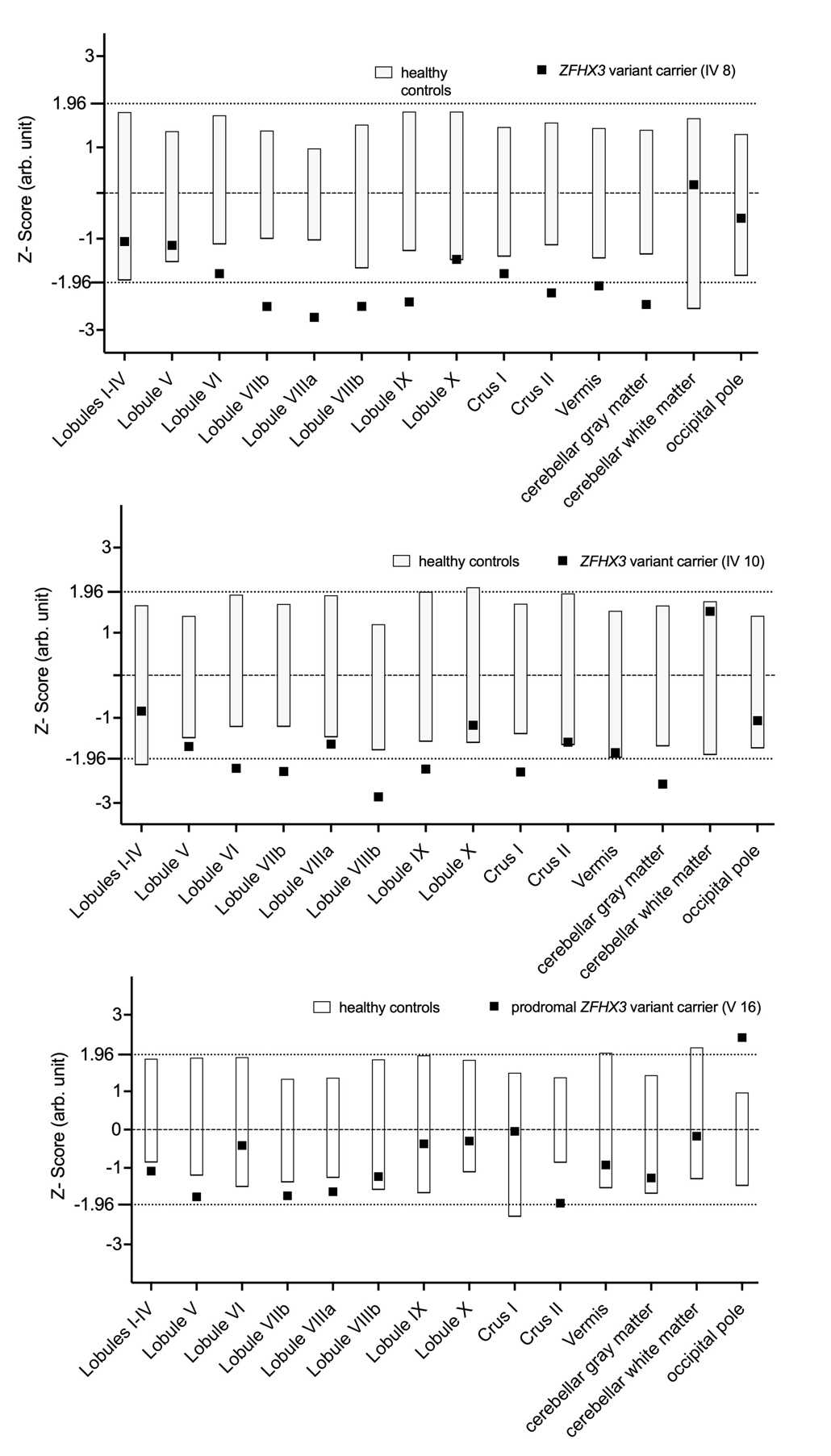


Supp. Fig 2: Results of cerebellar volumetry: The figure shows TIV-standardized ROI-volumes of each *ZFHX3* variant carrier (black rectangular dot) compared to their respective control group (bars). All volumes are z-transformed for comparability. The occipital lobe was chosen a control region. arb. units, arbitrary units; ROI, region of interest; TIV, total intracranial volume.
